# Expression of AIM2 is high and correlated with inflammation in hepatitis B virus associated glomerulonephritis

**DOI:** 10.1186/1476-9255-10-37

**Published:** 2013-12-10

**Authors:** Wenjun Du, Junhui Zhen, Zhaomin Zheng, Shumin Ma, Shijun Chen

**Affiliations:** 1 Shandong University School of Medicine, Jinan, China; 2 Digestive Department, Shandong provincial Qianfoshan hospital, Shandong University, Jinan, China; 3 Department of Pathology, Shandong University School of Medicine, Jinan, China; 4 Department of Liver Disease, Jinan Infectious Disease Hospital, Shandong University School of Medicine, Jinan, China

**Keywords:** Hepatitis B virus associated glomerulonephritis, Chronic glomerulonephritis, Absent in melanoma 2, Caspase-1, Interleukin-1β

## Abstract

**Background & aims:**

Innate immunity is the first line of defense against invasive microbial infection, and AIM2 plays an important role in this process by sensing double-stranded DNA viruses. However, the role of AIM2 in regulating the immune response to viruses in vivo, especially in sensing hepatitis B virus (HBV), has not been examined. We hypothesized that the expression of AIM2 increases corresponding to HBV-mediated inflammation in patients with hepatitis B virus associated glomerulonephritis (HBV-GN), a condition which activates inflammatory mechanisms and causes renal damage. To test this hypothesis, we analyzed the expression of AIM2 in HBV-GN patients in relation to the inflammatory response to HBV infection.

**Methods:**

A total of 79 patients diagnosed with chronic nephritis (CN) were enrolled in this study, including 54 HBV-GN patients as the experimental group and 24 chronic glomerulonephritis (CGN) patients as the negative control group. Six patients diagnosed with chronic hepatitis B (CHB) were also enrolled as positive controls. Each CN patient received renal biopsy, and immunohistochemistry was used to detect the expression of AIM2 and inflammatory factors caspase-1 and IL-1β in the biopsy specimens. CHB patients received liver puncture biopsy, and immunohistochemistry was used to detect the expression of AIM2 in these specimens. Expression of AIM 2 among different groups and in relation to inflammatory factors caspase-1 and IL-1β was analyzed.

**Results:**

The expression of AIM2 in HBV-GN patients (81.4%) was significantly higher than in CGN patients (4.0%). Among the HBV-GN patients, expression of AIM2 was significantly higher in the high HBV replication group than in the low HBV replication group. AIM2 expression was not correlated with age, gender, HBeAg status in serum, HBV-antigen type deposited in renal tissue or pathological type of HBV-GN. However, AIM2 levels were positively correlated with the expression of caspase-1 and IL-1β in HBV-GN patients. The data suggest that AIM2 expression is directly correlated with HBV infection-associated inflammation.

**Conclusion:**

The elevation of AIM2 during HBV infection or replication may contribute to its associated inflammatory damage, thus providing a putative therapeutic target and a new avenue for researching the pathogenesis of HBV-GN.

## Introduction

Hepatitis B virus (HBV) infection has been shown to induce several extra-hepatic lesions [1], One of the most common manifestations is hepatitis B virus associated glomerulonephritis (HBV-GN) [2]. Since the association between HBV infection and glomerular diseases was first reported by Combes et al. in 1971 [3], more HBV-GN cases have been described all over the world. The existence of HBV DNA in the renal tissue of some nephritic patients led to the classification of HBV-GN, proposing a role for HBV in its pathogenesis [4]. However, the specific pathogenesis of HBV-GN is still unclear. The widely accepted view is that persistent viral infections could lead to immune complex-mediated nephritis [5].

Absent in melanoma 2 (AIM2) is a member of the HIN-200 protein family [6] and can bind to double-stranded DNA (ds-DNA) and to the adaptor molecule ASC (apoptosis-associated speck-like protein), which contains a caspase activation and recruitment domain. This complex then activates caspase-1 and leads to the formation of mature IL-1β. An important intracellular inflammatory cytokine, maturation and secretion of IL-1β causes subsequent tissue damage [7]. Although AIM2 is known to be involved in the host defense against microbial invasion, its role in regulating the immune response to viruses, especial to HBV, has not been well understood. HBV is a ds-DNA virus, and HBV particles have been shown to be detectable in the kidneys of HBV-GN patients [4]. Cytoplasmic HBV-DNA in the kidneys has a chance to be recognized by AIM2. The potential binding of AIM2 to HBV-DNA may lead to the activation of caspase-1 and subsequent maturation and secretion of IL-1β. This cascade of events leads to the development of the inflammasome, which may then be responsible for the renal damage seen in HBV-GN patients.

In this study, we compared the expression of AIM2 in HBV-GN and chronic glomerulonephritis (CGN) and further explored the relationship between the expression of AIM2, caspase-1 and IL-1β. Our results showed that AIM2 expression is high in HBV-GN and correlated with both serum HBV-DNA titer and renal inflammation associated with HBV-GN, thus AIM2 may play an important role in the development and progression of inflammation.

## Materials and methods

### Patients

Our retrospective study was approved by the ethics committee of Jinan Infectious Disease Hospital. A total of 79 patients diagnosed with chronic nephritis, identified between 2008 and 2011 at Jinan Infectious Disease Hospital and QiLu Hospital of Shandong University Shandong, China, were included in the study. The experimental group consisted of 54 HBV-GN patients, the negative control group consisted of 25 CGN patients, and six chronic hepatitis B (CHB) patients served as the positive control group. The six CHB patients were serum HBeAg-positive and had HBV-DNA titers of more than 1 × 10^5^ copies/ml. Subjects received either kidney (CGN) or liver (CHB) puncture biopsy under ultrasound guidance to attain nephridial tissue or hepatic tissue, respectively, for diagnosis and subsequent research. Participation was dependent upon fulfillment of the following criteria: (1) patients must not have used an immune agent or anti-viral agent in the past three months; (2) patients must not have HAV, HCV, HDV, HEV or HIV co-infection; (3) patients must not have a history or current evidence of secondary glomerulonephritis; and (4) consent for participation must have been obtained from those who participated.

### Diagnosis of HBV-GN, CGN and CHB

**Figure 1:**
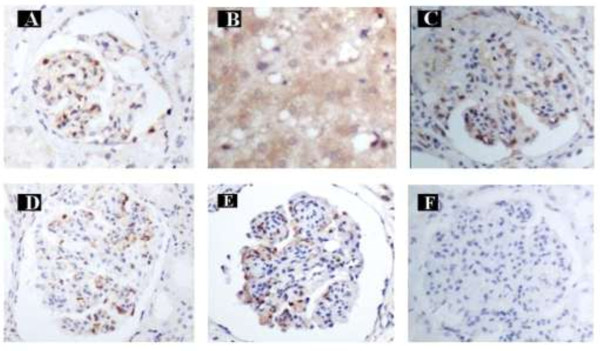
**Immunohistochemical staining. A**: HBsAg positive staining in glomerular endothelial cells and mesangial cells in HBV-GN (Magnification of 400x). **B**: AIM2 positive staining in hepatic cytoplasm in CHB (Magnification of 400x). **C**: AIM2 positive staining in glomerular endothelial cells and mesangial cells in HBV-GN (Magnification of 400x). **D**: Caspase-1 positive staining in glomerular endothelial cells and mesangial cells in HBV-GN (Magnification of 400x). **E**: IL-1β positive staining in glomerular endothelial cells and mesangial cells in HBV-GN (Magnification of 400x). **F**: AIM2, Caspase-1 and IL-1β negative staining in glomerular endothelial cells and mesangial cells in CGN (Magnification of 400x).

The diagnostic criteria used for CGN and HBV-GN were in accordance with the 2002 Kidney Disease Outcome Quality Initiative (K/DOQI), edited by the National Kidney Foundation (NKF) [8]. The diagnosis of HBV-GN was confirmed by pathology. Frozen slices from biopsies of the 54 HBV-GN patients were kept in a low-temperature freezer. Monoclonal goat-anti-human HBsAg and HBcAg antibodies were purchased from Dako (Denmark), and immunohistochemical staining for HBsAg and HBcAg in renal biopsies was used to confirm the diagnosis (Figure 1A). For HBV-GN patients with undetectable HBsAg or HBcAg in nephridial tissue, HBV was detected using the JCM-6000 scanning electron microscope from Jeol, Ltd. (Japan).

The diagnostic criteria used for CHB were in accordance with the Asian-Pacific Consensus Statement on the Management of Chronic Hepatitis B [9].

### The pathological classification of HBV-GN

The pathological classification of and diagnostic criteria used for HBV-GN were in accordance with 1990 WHO classification criteria [10]. Sections from all biopsy specimens were stained routinely with hematoxylin and eosin (H&E), periodic acid-sliver methenamine (PASM), Masson’s trichrome and antibodies against IgA, IgG, IgM, C3 and C1q complement component. Fluorescently-labeled IgA, IgG, IgM, C3 and C1q rabbit-anti-human antibodies were purchased from Dako.

### Immunohistochemistry and scoring

Nephridial and hepatic tissue specimens were first fixed in 10% formalin, then the tissue was cut, dehydrated, dipped in wax, embedded and sectioned. These sections were then placed on slides, baked, placed into xylene, cleared of the wax, rehydrated using graded ethanol and immersed in 0.3% hydrogen peroxide for five minutes to reduce non-specific background staining caused by endogenous peroxidase. The slides were then washed with PBS buffer three times for five minutes each, placed in citrate buffer solution at a pH of 6.0 and then into a high temperature pressure pot to recover the tissue antigen. After being heated, the slides were cooled and restored at room temperature, washed three more times in PBS buffer and incubated with AIM2 (ab93015, rabbit anti-human polyclonal antibody; Abcam, USA), caspase-1 (sc-56063,mouse anti-human polyclonal antibody; Santa Cruz Biotechnology Inc., USA) and IL-1β antibodies (ab2105, rabbit anti-human polyclonal antibody; Abcam), respectively. The slides were then placed in a 4°C refrigerator overnight. The next day, the slides were washed with PBS buffer three times, each time lasting longer than five minutes, then incubated with the secondary antibody PV-9000 (universal antibody) at 37°C for 10 minutes, washed with PBS buffer, and DAB staining was applied. The stain was terminated using running water, then the slides were washed with hydrochloric acid alcohol for differentiation. Lastly, the slides were washed with distilled water, cleared with xylene and mounted.

Appearance of a tan stain in the cytoplasm signaled positive expression of the protein. After staining, scores were assigned based on stain intensity and percentage of positive cells as follows: For stain intensity, a score of 0 was given for no brown staining (i.e., no cells stained), 1 for light brown, 2 for brown and 3 for dark brown; for percentage of positive cells, a score of 0 was given for fewer than 5% positive cells, 1 for 5% to 30%, 2 for 30% to 60% and 3 for greater than 60%. Scores for stain intensity and percent positive were then added together, and a negative sign (−) was assigned for scores totaling 0, mildly positive (+) for scores between 1 and 3, moderately positive (++) for scores between 4 and 6 and strongly positive (+++) for scores greater than 7.

### Statistical analysis

The SPSS program (version 17.0) was used for analysis. Measurement data was described as mean ± standard deviation. Background factors were compared using the Student’s t-test (numerical data) or the Chi-square test (categorical data). Spearman’s two-tailed test was used for correlation analysis, and differences were regarded as significant if the *p* value was less than 0.05 on either side.

## Results

### Expression of AIM2 was significantly high and correlated with HBV load in HBV-GN

To study the role of AIM2 in HBV-GN, we compared the expression of AIM2 in 54 HBV-GN and 25 CGN patients. Expression of AIM2 in the six positive control CHB patient liver specimens was confirmed by immunohistochemistry. The results showed that AIM2 expression was exclusive to the hepatic cellular cytoplasm in liver tissue (Figure 1B) and the cellar cytoplasm of glomerular endothelial cells and mesangial cells in nephridial tissue. Statistical analysis revealed that the positive expression rate of AIM2 in HBV-GN patients was significantly higher than in CGN patients (81.4% vs. 4.0%, *p* < 0.01) (Table 1). Notably, AIM2 expression was not affected by age (*p* = 0.06) or gender (*p* = 0.527).

**Table 1:** The expression of AIM2 is high in HBVGN

**Group**	**Tissue**	**n**	**Age**	**Gender M(%)**	**AIM2**				**Positive rate(%)**
					**-**	**+**	**++**	**+++**	
HBV-GN	K	54	36.1 ± 12.7	35(64.8)	10	26	18	0	81.4
CGN	K	25	38.2 ± 15.5*	18(72.0)**	24	1	0	0	4.0***
CHB	L	6	33.0 ± 8.7	6(100)	0	0	6	0	-

HBV-GN, Hepatitis B viral associated glomerulonephritis; CGN, Chronic glomerulonephritis; CHB, Chronic hepatitis B; K, kidney, L, liver; *compared with HBV-GN, (t = −1.909, *p* = 0.06); **compared with HBV-GN, (x^2^ = 0.400, *p* = 0.527); ***compared with HBV-GN, (x^2^ = 38.746, *p* < 0.01).

To further clarify the factors affecting the expression of AIM2 in HBV-GN patients, we considered the potential influence of age, gender, HBeAg status and HBV titer in serum. As summarized in Table 2, the results showed that AIM2 expression was not affected by age (*p =* 0.937) or gender (*p =* 0.627). Using ELISA, serum HBeAg detected in 47 of the 54 HBV-GN patients showed that AIM2 expression was also not affected by HBeAg status (*p =* 0.614). However, in assessing HBV titers, real-time PCR detected serum HBV-DNA in 24 of 54 tested HBV-GN patients, and we found that the expression of AIM2 was significantly higher in patients with high viral load (HBV-DNA ≥ 1 × 10^5^copies/ml) than in patients with low viral load (HBV-DNA < 1 × 10^5^ copies/ml) (*p* < 0.05). These results suggested that the expression of AIM2 may be dependent on HBV load in HBV-GN.

**Table 2:** Expression of AIM2 was correlated with serum HBV load in HBV-GN

		**AIM2**				
	**n(%)**	**-**	**+**	**++**	***X***^***2***^	***p***
Gender, M	35(64.8%)				0.131	0.937
Age (y)					2.598	0.627
≤20	5(9.3%)	2(20.0%)	2(7.7%)	1(5.6%)		
21-40	29(53.7%)	4(40.0%)	16(61.5%)	9(50.0%)		
≥41	20(37.0%)	4(40.0%)	8(30.8%)	8(44.4%)		
e-Ag (+)	33(70.2%)	6(60.0%)	16(69.6%)	11(78.6%)	0.975	0.614
HBV-DNA						
≥10^5^ cp/ml	16(66.7%)	5(83.3%)	10(76.9%)	1(20.0%)	6.097	0.047

M, male; e-Ag, HBeAg; cp/ml, copies/ml.

### Expression of AIM2 is not correlated with HBV-antigen type deposited in nephridial tissue or with pathological type of HBV-GN

To test whether the expression of AIM2 may be influenced by various HBV-antigen types deposited in nephridial tissue, we compared the expression of AIM2 among groups with various HBV antigens deposited in nephridial tissue. As summarized in Table 3, the results showed no difference in the expression of AIM2 among various HBV-antigen groups (*p* = 0.511).

**Table 3:** Expression of AIM2 was negatively correlated with various HBV antigen deposited in kidney tissue in HBV-GN

		**AIM2**				
	**n(%)**	**-**	**+**	**++**	***X***^***2***^	***p***
s-Ag+,c-Ag+	22(40.7)	5	10	7		
s-Ag+,c-Ag-	24(44.4)	4	12	8		
s-Ag-,c-Ag+	6(11.1)	1	4	1		
s-Ag-,c-Ag-	2(3.7)	0	0	2		
Total	54	10	26	18	5.259	0.511

s-Ag = HBsAg; c-Ag = HBcAg.

We also analyzed the expression of AIM2 among various pathological types of HBV-GN. As summarized in Table 4, the results showed no difference in the expression of AIM2 among various pathological types of HBV-GN (*p* = 0.940).

**Table 4:** Expression of AIM2 was negatively correlated with various pathological types of HBV-GN

		**AIM2**				
**Pathological type**	**n(%)**	**-**	**+**	**++**	***X***^***2***^	***p***
MsPGN	15(27.8)	3	8	4		
MPGN	9(16.7)	1	5	3		
MN	27(50.0)	6	11	10		
MCG	1(1.8)	0	1	0		
FSS	2(3.7)	0	1	1		
Total	54	10	26	18	2.905	0.940

MsPGN, Mesangioproliferative glomerulonephritis; MPGN, Membranoproliferative glomerulonephritis; MN, Membranous nephropathy; MCG, Minimal change glomerulopathy; FSS, Focal segmental sclerosis.

### Expression of AIM2 was correlated with inflammation in HBV-GN

IL-1β is widely accepted to be an important pro-inflammatory factor contributing to the development of chronic renal inflammation. To reveal what role AIM2 plays in HBV-GN patient renal damage, we analyzed the correlation between the expression of AIM2, caspase-1 and IL-1β. Statistical analysis revealed that the expression of AIM2 was positively correlated with that of caspase-1 (rs = 0.444, *p* < 0.01) and IL-1β (rs = 0.379, *p* < 0.01), and expression of caspase-1 was positively correlated with that of IL-1β (rs = 0.515, *p* < 0.01) (Table 5). Figure 1C, D and E illustrate the positive expression of AIM2, caspase-1 and IL-1β, respectively, in these patients, and Figure 1F shows the negative expression of AIM2 in CGN. These results suggested that the expression of AIM2 was specifically correlated with inflammation in HBV-GN and that elevation of AIM2 corresponding to HBV infection or replication may contribute to the inflammatory damage associated with the development of HBV-GN.

**Table 5:** Expression of AIM2 was positively correlated with caspase-1 and IL-1β in HBV-GN

**AIM2**	**Caspase-1**				**IL-1β**			
	**-**	**+**	**++**	**+++**	**-**	**+**	**++**	**+++**
-	7	2	1	0	11	19	4	0
+	4	19	3	0	4	16	7	0
++	2	9	6	1	3	6	8	1

AIM2 was positively correlated with caspase-1 (rs = 0.444, *p* < 0.01); AIM2 was positively correlated with IL-1β (rs = 0.379, *p* < 0.01); caspase-1 was positively correlated with IL-1β (rs = 0.515, *p* < 0.01).

## Discussion

AIM2 was first reported to act as a putative tumor suppressor in malignant melanoma [11]. Recent research demonstrated that AIM2 plays an important role in the innate immune response through sensing potentially dangerous cytoplasmic ds-DNA and inducing the formation of the ASC inflammasome, which then induces the activation of caspase-1 and release of mature IL-1β [7]. Maturation and secretion of IL-1β, an important intracellular cytokine belonging to the IL-1 superfamily [12], ultimately leads to tissue damage [13]. As a non-specific receptor for cytoplasmic DNA, AIM2 can be activated by and bind to plasmid DNA, DNA from the bacterium *Listeria monocytogenes* and even synthetic ds-DNA [14]. Previous research detected AIM2 in the small intestine, spleen, peripheral white blood cells and testis [15]. Our study describes the inducible expression of AIM2 in liver and kidney tissue and the exclusive expression of AIM2 in the hepatic cytoplasm and glomerular endothelial cell and mesangial cell cytoplasm in the kidney. These results further suggest that AIM2 may play an important inflammatory role in not only HBV-GN but also CHB and may provide a new avenue for researching the pathogenesis of CHB. We found that the positive expression rate of AIM2 in the HBV-GN group was significantly higher than that of the CGN group. As neither age nor gender were statistically different between the two groups, this suggests that there is indeed a relationship between chronic HBV infection and AIM2 elevation. We also considered another potential influence factor, HBeAg status in serum, which may have contributed to the mutation of the HBV-DNA-P-BCP (basal core promoter) or pre-C region, influencing AIM2 activation and binding to HBV-DNA. Our results showed that the expression of AIM2 was not significantly different between the HBeAg positive and negative groups, demonstrating that AIM2 activation and binding to HBV-DNA is not influenced by serum HBeAg status. Moreover, our results indicated that the positive expression rate of AIM2 in the high replication group (HBV-DNA ≥ 1 × 10^5^ copies/ml) was significantly higher than that of the low replication group (HBV-DNA < 1 × 10^5^ copies/ml), suggesting that high HBV load led to the increase in AIM2 expression. A recent study focusing on the relationship between AIM2 expression and acute and chronic hepatitis B infection showed that AIM2 mRNA expression was negatively correlated with serum HBV load [16]. This study included acute hepatitis B (AHB) and chronic hepatitis B (CHB) patients. The pathogenesis of AHB and CHB is widely accepted to be high HBV load assault and immune injury, respectively, though the pathogenesis of each in the study was unclear. For both AHB and CHB, only inflammation (i.e., immune injury) was noted. The role of AIM2 in regulating the immune response to viral infection can hereby be emphasized. A higher HBV load provides a greater opportunity to bind AIM2 and lead, subsequently, to activation of the inflammation signal pathway. However, some of the CHB patients in the characteristic stages of immune tolerance, immune clearance, inactive-carrier and reactivation in this study exhibited no inflammation. In our present study, all HBV-GN patients exhibited inflammation. Moreover, the pathogenesis of CHB and HBV-GN is different. As for the role of HBV in HBV-GN pathogenesis, it needs to be explored further. Previous research reported that high HBV-DNA load was correlated with HBV-GN-related morbidity [4]; however, the specific pathogenesis for this has been unclear until now. Our results have demonstrated that this morbidity may be due to the high HBV-DNA load of these patients having a greater chance to activate AIM2, resulting in the release of inflammatory cytokines and subsequent renal damage.

Several HBV antigens, including HBsAg and HBcAg, have been found deposited in the glomerulus and thought to contribute to the pathogenesis of HBV-GN [17]. Our data indicate that the expression of AIM2 is not correlated with the type of HBV-antigen deposited in nephridial tissue, suggesting also that AIM2 binding to HBV-DNA was not dependent on HBV-antigen type deposited. Previous research demonstrated that different pathological types of HBV-GN have different clinical outcomes [18], so we further analyzed the expression of AIM2 among various pathological types within the HBV-GN group. The results demonstrated that the expression of AIM2 was not affected by these various pathological types of HBV-GN, suggesting that inflammation due to AIM2 activation commonly exists irrespective of pathological type.

IL-1β is an important intracellular cytokine which binds to receptors and activates the downstream NF-ĸB signaling pathway, releasing inflammatory factors that cause tissue damage. Our results showed that the expression of AIM2 was positively correlated with the expression of caspase-1 and IL-1β in HBV-GN. HBV particles were detectable using scanning electron microscopy in nephridial tissue from HBV-GN patients [4]. During the innate immune response to invading HBV, activated AIM2 may bind to ASC, inducing activation of caspase-1 and releasing IL-1β [7]. Similar results were obtained in the recent Wu study focusing on the relationship between AIM2 expression and acute and chronic hepatitis B infection [16], suggesting that AIM2 plays an important role in both HBV-GN and hepatitis B infection. As long as HBV is present, AIM2 may be activated and lead to subsequent inflammatory injury. Our study further confirms this mode of signal transduction. Within the experimental group, the expression level of AIM2 was positively correlated with the level of caspase-1, and the level of caspase-1 was positively correlated with the level of IL-1β, suggesting that this inflammation signal transfer pathway was related to AIM2 levels in HBV-GN and, subsequently, that AIM2 may play an important role in the pathogenesis of HBV-GN.

In summary, we found the expression of AIM2 to be significantly increased in HBV-GN patients. AIM2 levels were highly correlated with HBV load and inflammation in HBV-GN. Collectively, these results suggest that the elevation of AIM2 during HBV infection or replication may contribute to renal damage due to inflammation. Our findings may help provide a new therapeutic target for HBV-GN and a new avenue for researching its pathogenesis.

## Abbreviations

HBV: Hepatitis B virus; HBV-GN: Hepatitis B virus associated glomerulonephritis; AIM2: Absent in melanoma 2; ASC: Apoptosis-associated speck-like protein; IL-1β: Interleukin-1β; CHB: Chronic hepatitis B; HAV: Hepatitis A virus; HCV: Hepatitis C virus; HEV: Hepatitis E virus; HIV: Human immunodeficiency virus; K/DOQI: Kidney Disease outcome quality initiative; NKF: National Kidney Foundation; H&E: Hematoxylin and eosin; PASM: Periodic acid-sliver methenamine; MsPGN: Mesangioproliferative glomerulonephritis; MPGN: Membranoproliferative glomerulonephritis; MN: Membranous nephropathy; MCG: Minimal change glomerulopathy; FSS: Focal segmental sclerosis; BCP: Basal core promoter.

## Competing interests

None of the authors has an affiliation or conflict of interests.

## Authors’ contributions

Study concept and design: CSJ, Acquisition of data: DWJ and ZJH, Analysis and interpretation of data: DWJ and ZZM, Drafting of the manuscript: DWJ, MSM and ZJH, Critical revision of the manuscript for important intellectual content: CSJ, Statistical analysis: DWJ and MSM, Administrative, technical or material support: ZJH, ZZM and MSM, Study supervision: CSJ. All authors read and approved the final manuscript.
